# Oncological characteristics, treatments and prognostic outcomes in MMR-deficient colorectal cancer

**DOI:** 10.1186/s40364-024-00640-7

**Published:** 2024-08-26

**Authors:** Wen-Xuan Fan, Fei Su, Yan Zhang, Xiao-Ling Zhang, Yun-Yi Du, Yang-Jun Gao, Wei-Ling Li, Wen-Qing Hu, Jun Zhao

**Affiliations:** 1 Graduate School of Shanxi Medical University, Taiyuan, Shanxi 030607 China; 2 Department of Oncology, Changzhi People’s Hospital Affiliated to Changzhi Medical College, Changzhi, Shanxi 046000 China; 3 Graduate School of Changzhi Medical College, Changzhi, Shanxi 046000 China; 4 Department of Gastrointestinal Surgery, Changzhi People’s Hospital Affiliated to Changzhi Medical College, Changzhi, Shanxi 046000 China

**Keywords:** dMMR colorectal cancer, Microsatellite instability, Lynch syndrome, Lynch-Like syndrome, Treatment, Prognosis

## Abstract

Colorectal cancer (CRC) ranks as the third most prevalent cancer globally. It’s recognized that the molecular subtype of CRC, characterized by mismatch repair deficiency (dMMR) or microsatellite instability-high (MSI-H), plays a critical role in determining appropriate treatment strategies. This review examines the current molecular classifications, focusing on dMMR/MSI-H CRC and its subtypes: Lynch syndrome (LS), Lynch-like syndrome (LLS), and sporadic cases. Despite advances in understanding of these genetic backgrounds, clinical trials have not conclusively differentiated the efficacy of immune checkpoint inhibitors among these subgroups. Therefore, while this review details the molecular characteristics and their general implications for treatment and prognosis, it also highlights the limitations and the need for more refined clinical studies to ascertain tailored therapeutic strategies for each subtype. Furthermore, this review summarizes completed and ongoing clinical studies, emphasizing the importance of developing treatments aligned more closely with molecular profiles. By discussing these aspects, the review seeks to provide a comprehensive analysis of oncological characteristics, presenting a detailed understanding of their implications for treatment and prognosis in dMMR/MSI-H CRC.

## Introduction

Colorectal cancer (CRC) currently accounts for 9.7% of cancer incidences and 9.4% of cancer-related deaths worldwide [1, 2]. In China, the average 5-year survival rate for CRC is 56.9% [3], ranking second in global cancer-related mortality [4]. Recently, the latest statistical report on the incidence and mortality of CRC in the United States indicated that while the overall incidence and mortality rates of CRC are decreasing, the disease burden is rapidly shifting towards younger patients and a growing trend of more individuals progressing to advanced stages [5]. Neoadjuvant therapy, surgery, adjuvant treatment, radiation therapy, and chemotherapy are the main treatment methods for CRC patients, but they may lead to changes in the patient’s physical appearance, varying degrees of impairment in basic physical functions, and social functional impairments [6, 7].

Microsatellites, also known as short tandem repeats, are DNA sequences in the genome consisting of a small number of nucleotide repeats. In cases where there is a malfunction in the DNA mismatch repair (MMR) system, errors in the replication of microsatellites accumulate, leading to changes in the length or composition of the sequences, a condition known as microsatellite instability (MSI). This instability gives rise to a high mutation phenotype [8]. Deficient MMR (dMMR) in tumors is usually caused by pathogenic mutations in MMR genes such as mutL homolog 1 (MLH1), muts homolog 2 (MSH2), muts homolog 6 (MSH6), postmeiotic segregation increased 2 (PMS2), or in the epithelial cell adhesion molecule (EPCAM) gene. It can also be caused by high methylation of the MLH1 promoter region, leading to loss of MLH1 expression [9]. MSI is classified into three categories: high instability (MSI-H), low instability (MSI-L), and stable (MSS) [10]. The National Comprehensive Cancer Network (NCCN) recommends the use of the 2B3D NCI panel and 5 mononucleotide marker panel primarily based on the Promega Panel for MSI testing [11, 12]. In stage II CRC, approximately 20% of patients exhibit dMMR/MSI-H, while in stage III, this proportion is around 12%. The prevalence is lowest in stage IV, at approximately 4% [13]. The prevalence of dMMR/MSI-H CRC varies by ethnicity [14, 15]. Studies has reported higher rates of MSI-H CRC among Europeans (5-24%) and Caucasian Americans of European descent (8-20%), as well as African Americans (12-45%) and Egyptians (37%) [8, 16–18]. In contrast, MSI-H CRC prevalence is relatively lower in Asian countries, with rates of 4.5-15.0% in China and 3.8 − 20.0% in Japan [19, 20].

**Fig. 1:**
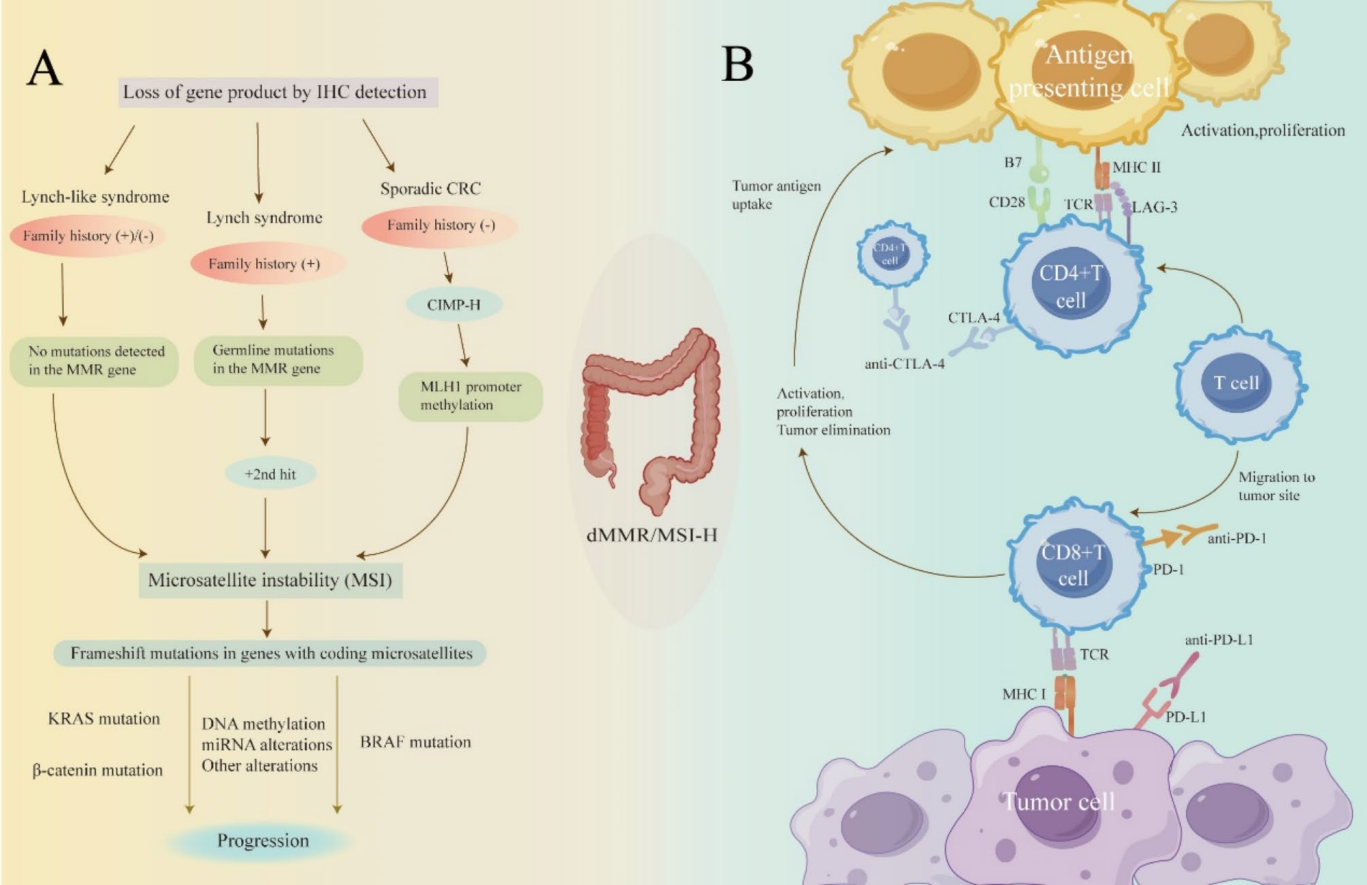
**(A)** LLS tumors exhibit MSI or MMR expression deficiency (without BRAF mutation and MLH1 methylation), with no germline MMR mutations. Patients with LLS can be categorized into those with a family history and those without a cancer history. LS tumors are caused by germline mutations in MMR genes, followed by a second hit on the wild-type copy, which may occur through LOH, methylation, or point mutations, often accompanied by KRAS mutation and a family history of CRC. Sporadic CRC typically lack a family history and exhibit CpG island methylator phenotype (CIMP) characteristics, leading to methylation of numerous gene promoters. When the MLH1 promoter is methylated, MMR activity is disrupted, leading to MSI, with BRAF mutations observed in the majority of sporadic colorectal tumors, but not in tumors of LS patients. **(B)** PD-L1 is a ligand of PD-1 and is associated with immune suppression. The expression of PD-L1 by tumor cells can lead to T cell evasion, enabling immune escape. Anti-CTLA-4 antibodies activate anti-tumor immunity, while LAG-3 inhibits activated T cells, terminating signal transduction after binding to class II MHC molecules and class I MHC molecules, leading to a decline in immune response

The diagnostic and treatment approaches for advanced CRC patients with pMMR/MSS and dMMR/MSI-H subtypes are completely different, leading to distinct therapeutic regimens and varying treatment outcomes. dMMR/MSI-H CRC commonly presents in three clinical types: Lynch syndrome (LS), Lynch-like syndrome (LLS) and sporadic CRC (Fig. 1A). This article aims to delineate the molecular characteristics and their general implications for treatment, and prognosis of these three types.

## Molecular biological characteristics of dMMR/MSI-H

### Molecular mechanism and genetic background of 3 types of dMMR/MSI-H CRC

Hereditary nonpolyposis colorectal cancer (HNPCC), also known as LS, is a common autosomal dominant hereditary tumor syndrome, accounting for approximately 2–4% of CRCs [21]. The Amsterdam I and II criteria, the Bethesda guidelines, and the revised Bethesda guidelines are all screening criteria specifically for LS. They are used to identify CRC patients who require dMMR or MSI testing.LS is mainly caused by germline mutations in the MLH1, MSH2, MSH6, or PMS2 genes, or by deletion of the EPCAM gene resulting in non-expression of MSH2 [22]. The “second hit” associated with these mutations leads to the inactivation of the other allele, resulting in tumor development. Tumors often present with mismatch repair deficiencies [23, 24].

Lynch-like syndrome (LLS) refers to patients who present with features resembling LS, such as DNA mismatch repair deficiency, without detectable mutations in the genes encoding MMR proteins. LLS CRC cases present with MSI and deletion of MLH1, MSH2, MSH6 or PMS2 expression, as detected by immunohistochemistry (IHC) [25–27]. Unlike sporadic CRC, LLS does not display epigenetic silencing of MLH1 or mutations in the B-Raf proto-oncogene, serine/threonine kinase (BRAF). Studies have suggested that patients with LLS may harbor germline mutations in non-DNA MMR genes associated with the molecular mechanisms of LS, such as in the POLD1/POLE DNA polymerase genes [28].

The MSI is observed in approximately 15% of all sporadic CRC. MSI tumors are more common in the elderly (≥ 70 years) and are often found in women [29, 30]. Somatic mutation and epigenetic hypermethylation of the MLH1 gene promoter region are the main causes of sporadic MSI-H CRC, leading to transcriptional silencing [31, 32], The presence of the BRAFV600E is found in sporadic MSI CRC, and BRAFV600E is an activating mutation that leads to sustained mitogen-activated protein kinase (MAPK) pathway signalling [33, 34]. The high CpG island methylator phenotype (CIMP-H) refers to the high methylation status of three or five specific genes and is associated with MLH1 promoter methylation and MSI-H. Although CIMP-H patients show no response to 5-fluorouracil chemotherapy, those with CIMP-H and MSI have a higher survival rate [35].

LLS patients tend to be younger at onset, and the family history of CRC incidence is intermediate between LS and sporadic CRC [28], Among LLS CRC patients, 50-60% exhibit biallelic somatic inactivation of genes [36, 37]. LLS patients can be divided into two groups (Fig. 1A): those with a family history (potentially involving unknown hereditary risk factors) and those without a history of cancer (possibly harboring biallelic somatic mutations in MMR genes), the latter being considered as sporadic CRC [38, 39]. According to the revised Bethesda guidelines, a positive family history alone does not necessarily indicate a hereditary case; however, if the type of family history meets the Amsterdam criteria, it may suggest a hereditary case. Currently, there is no consensus on whether LLS should be classified as a potential hereditary or sporadic condition, as LLS patients may represent a heterogeneous group [40]. Although the use of multi-gene testing in LLS has increased in recent years, the optimal diagnostic strategy for LLS patients has not yet been established. Therefore, expert consensus is needed to accurately classify these cases as either true sporadic or potentially hereditary [38, 41]. Family history, age of onset, and prognosis are key clinical features distinguishing LLS patients from those with sporadic MSI-H CRC. Therefore, LLS cases encompass both sporadic and hereditary cases, and it is necessary to define molecular tools to effectively distinguish between the two groups [36].

Distinguishing between sporadic CRC patients and LS or LLS patients can be achieved through the age of CRC onset, a history of cancer in the family, and the presence of BRAFV600E and/or MLH1 methylation. Additionally, despite the lesion location being similar to LS, the presence of MLH1 hypermethylation and BRAFV600E in patients with sessile serrated adenoma/polyps (SSA) suggests that SSA is not part of LS [42].

### dMMR/MSI-H CRC detection techniques

Current techniques used for MSI detection include IHC, PCR, NGS, and b-MSI assays [43].

The IHC detection of MMR protein loss and PCR amplification of microsatellite repeat sequences are widely used for clinical MSI screening. IHC is a simple and cost-effective method that has been widely used to detect protein expression. However, it cannot reflect gene variations and its screening utility is comparable to PCR analysis, with a consistency of 90.4-99.6% [44, 45]. IHC demonstrates a sensitivity of 74% for predicting MLH1 mutations and 91% for MSH2 mutations, alongside specificities of 81% and 90%, respectively. It effectively predicts the microsatellite instability (MSI) status in 76% of cases, with a specificity of 100% [46]. The interpretation criteria include the detection of four known MMR proteins (MLH1, MSH2, MSH6, and PMS2), with positive expression localized in the cell nucleus. If all four MMR proteins show positive expression, it is classified as pMMR. Any loss of expression of a MMR protein indicates dMMR, generally equivalent to the MSI-H phenotype.

The polymerase chain reaction (PCR) detection of MSI was the first established method for identifying MSI status in CRC and remains the current gold standard for MSI testing. The Bethesda panel, recommended by the National Cancer Institute in 1997, includes BAT-25, BAT-26, D5S346, D2S123, and D17S250. By 2002, this panel was revised to the Pentaplex panel, which added NR21 and NR24 to enhance sensitivity and specificity. In 2006, the MSI analysis system from Promega replaced NR27 with MONO-27, further increasing detection sensitivity. This panel has since been recognized by the National Cancer Institute as the international gold standard for identifying MSI in human tumors [47]. If two or more single nucleotide repeat fragment sizes change by ≥ 3 bp, the result is classified as MSI-H. Conversely, if only one fragment size changes by ≥ 3 bp or if no fragment size changes by > 3 bp, the result is classified as ‘non-MSI-H.’ It is important to highlight that studies have indicated that, in populations with high ethnic admixture, changes in a single nucleotide marker may correlate with the hereditary genetic characteristics of the individuals analyzed [48–51]. Recent reports have highlighted the high mutation frequency of the T17 single nucleotide repeat sequence in the gene encoding the chaperone HSP110 (HT17) in MSI CRC [52, 53]. Deletion of the HT17 repeat sequence in tumor DNA leads to exon 9 skipping (HSP110DE9), resulting in increased synthesis of HSP110 isoforms. Consequently, HT17 has been demonstrated as an improved biomarker for diagnosing MSI in CRC [54–56]. Despite its confirmed sensitivity, HT17 requires further evaluation, particularly in cancers beyond CRC.

Next-generation sequencing (NGS), as a second-generation sequencing method, provides comprehensive insights into genomic alterations and MSI status. Various algorithms using targeted, whole exome, or whole genome sequencing data have been designed to determine MSI status in NGS analysis [57–59], The NCCN guidelines recommend using validated NGS Panels for MSI testing, such as the FDA-approved MSK-IMPACT and Foundation One CDx, which cover 1000 and 95 MSI loci, respectively. These are particularly suitable for metastatic CRC patients requiring RAS and BRAF gene typing [60].Despite multiple MSI algorithms, the lack of a unified standard has led to challenges in standardizing NGS MSI testing. Moreover, NGS testing is costly and time-consuming, and large PD(L)1 clinical trials tend to use PCR or IHC rather than NGS to assess MSI/MMR status [61].

A blood-based MSI (b-MSI) NGS algorithm based on circulating tumor DNA (ctDNA) has also emerged as a new option for MSI testing in advanced solid tumor patients with difficult or inadequate tumor tissue sampling [62]. However, it should not be routinely recommended at present and only serves as an alternative means to definitively determine MSI status in patients lacking tissue. The content covered by b-MSI-NGS testing includes b-MSI, blood-based tumor mutational burden (b-TMB), and multi-gene (including MMR) germline/somatic mutation status.

Previous studies have shown a discordance rate of 1–10% between MMR protein IHC and MSI testing [63–67]. The common inconsistency is IHC indicating MSH6 expression loss while MSI indicates MSS, which is attributed to functional overlap between MMR proteins. IHC results may be misinterpreted due to poor staining quality. Therefore, under standardized operations and error avoidance, any positive result from either test can serve as a basis for using immune checkpoint inhibitors in advanced cancer (Table 1) [68].

**Table 1:** Comparison of MSI detection techniques and their accuracy

	Detection technique	Sample quality requirements	Accuracy description
MMR	IHC	Relatively low sample quality required	Potential for misjudgment
MSI			
	PCR	Tumor proportion ≥ 20% required	Gold standard
	NGS	Tumor proportion ≥ 10% required	High accuracy
b-MSI			
	Liquid biopsy + NGS	ctDNA AFmax ≥ 0.1%	Less sensitive

MMR: mismatch repair, MSI: microsatellite instability, b-MSI: blood-based MSI, IHC: immunohistochemistry, PCR: polymerase chain reaction, NGS: next-generation sequencing, ctDNA: circulating tumor DNA

### dMMR/MSI-H CRC pathologic types

dMMR/MSI-H is most commonly found in stage II colon cancer. Studies have shown that CRC with dMMR/MSI-H has similar pathological features: (1) the primary tumor is often located in the right half of the colon; (2) low pathological stage; (3) presence of mucinous components; (4) tumor‑infiltrating lymphocytes; (5) absence of necrotic cellular debris and the presence of a Crohn‑like nodular infiltrate [69–71] .

It can be seen that patients with dMMR/MSI-H CRC exhibit diverse prognostic characteristics. In comparison to patients with pMMR/MSS tumors, those with dMMR/MSI-H CRC demonstrate longer disease-free survival (DFS) and overall survival (OS), possibly due to higher lymphocyte infiltration in dMMR/MSI-H tumors. dMMR/MSI-H CRC may have an inflamed tumor microenvironment (TME), which can increase sensitivity to immune checkpoint inhibitors (ICIs) [72]. Another important pathological characteristic of MSI-H CRC is the peritumoral lymphocytic reaction, manifested by the clustering of lymphocytes into follicles surrounded by stellate fibroblasts, with some forming germinal centers [73]. Studies show that sporadic MSI-H CRC and LS CRC differ in histology and morphology. LS often presents with lymphocyte infiltration, tumor cell dedifferentiation, and adenomas, while sporadic MSI-H CRC are characterized by increased cytoplasmic eosinophils, large vacuolated nuclei, mucin secretion, poor differentiation, high heterogeneity, Crohn’s-like reactions, and a higher prevalence of serrated and sawtooth-like polyps [74, 75].And the LS patients typically exhibit more pronounced local T-cell infiltration and even higher mutational burden compared to sporadic dMMR/MSI-H CRC, leading to a better prognosis [76, 77].

In summary, different MSI statuses in CRC have specific clinicopathological features. Patients with dMMR/MSI-H CRC exhibit both favorable and unfavorable prognostic characteristics. The distinct nature of dMMR/MSI-H CRC determines the need for personalized treatment.

## Advances in the treatment of dMMR/MSI-H CRC

The clinical trials of dMMR/MSI-H CRC have not yet clearly distinguished the differences in the immunotherapy efficacy between LS, LLS, and sporadic patients, and lack unified classification criteria. Consequently, molecular-driven differentiated treatments cannot yet be provided, and treatment continues to follow the standard protocols for dMMR/MSI-H CRC. KRAS/BRAF, as independent prognostic indicators, guide the targeted therapy of CRC, but direct associations with immunotherapy have not been discovered. Preliminary research results indicate that the BRAFV600E mutation cannot predict the outcomes of dMMR/MSI-H metastatic CRC (mCRC) patients receiving ICI therapy [78]. Therefore, for mCRC patients with dMMR/MSI-H accompanied by BRAFV600E mutation, ICI should be considered standard first-line treatment [79].

For dMMR/MSI-H patients who are amenable to curative resection, phase II studies have shown that neoadjuvant immunotherapy can achieve an major pathological response (MPR) rate of 97-100% and a pathologic complete response (pCR) rate of 65%~88% [80–82]. In cases of partially T4b, M0 dMMR/MSI-H patients where curative resection cannot be achieved through combined organ excision, consideration may be given to the use of programmed cell death protein 1 (PD-1)/programmed death-ligand 1 (PD-L1) monoclonal antibodies, with pembrolizumab being the preferred option, as per the KEYNOTE-177 study and domestic indications for use in transformative or palliative therapy [83]. For dMMR/MSI-H stage III patients after curative surgery, studies related to the adjuvant therapy of ICIs are currently underway (Fig. 1B).

### Neoadjuvant therapy

In the CheckMate-142 study, combination immunotherapy achieved high objective response rates (ORRs) in both later-line and first-line treatment of advanced dMMR/MSI-H mCRC. This has prompted researchers to explore the use of immunotherapy for neoadjuvant treatment.

The NICHE study is the first clinical trial to explore the adjuvant treatment of early-stage colon cancer using dual immunotherapy. The study involved 20 dMMR and 20 pMMR patients. dMMR patients received combined treatment with nivolumab and ipilimumab, while pMMR patients were randomly assigned to receive dual immunotherapy with or without cetuximab. The study confirmed the safety and feasibility of PD-1 monoclonal antibody combined with low-dose cytotoxic T-lymphocyte-associated protein 4 (CTLA-4) monoclonal antibody therapy [80]. The NICHE-2 study [84] expanded the sample size of dMMR colon cancer patients, including 115 patients, with 111 participating in the study. These patients received combined neoadjuvant therapy with ipilimumab and nivolumab. The study results indicated that safety endpoints were met, with a MPR of 95% and a pCR of 68%. After a median follow-up of 13.1 months, no patients experienced recurrence [85]. Additionally, the results of the NICHE-3 study, presented at the 2023 ESMO Annual Meeting, demonstrated that using PD-1 monoclonal antibodies in combination with lymphocyte activation gene-3 (LAG-3) inhibitors as a dual adjuvant therapy for locally advanced dMMR colon adenocarcinoma patients achieved a pCR rate of 79% and a 100% pathological response rate [86]. These findings offer significant hope for adjuvant treatment of MSI-H/dMMR colon cancer patients, potentially realizing the goal of organ preservation and even drug-induced cure in the future.

The PICC study is a single-center, randomized, phase II clinical trial for patients with dMMR/MSI-H locally advanced CRC (LACRC), aiming to evaluate the efficacy of Trifluridine and Tipiracil Monotherapy or in combination with the cyclooxygenase-2 (COX-2) inhibitor Celecoxib as neoadjuvant therapy. The study confirmed that neoadjuvant therapy with Trifluridine and Tipiracil Monotherapy or in combination with Celecoxib is well tolerated and results in a high pCR rate [81].

The MDACC study is a phase II clinical trial of neoadjuvant therapy using pembrolizumab for dMMR/MSI-H locally advanced solid tumors, and the results showed a postoperative pCR rate of 83% and an MPR rate of 92% in CRC patients.

Pembrolizumab significantly improved the clinical outcomes of dMMR/MSI-H solid tumors, although there is still insufficient research on neoadjuvant therapy. A phase II study targeting locally unresectable or high-risk resectable dMMR/MSI-H tumors found that neoadjuvant pembrolizumab therapy is safe and effective, leading to a high pathological, radiological, and endoscopic response, which is of significant importance for organ preservation strategies [87].

The MSKCC study is a phase II clinical trial that explores the efficacy of the PD-1 inhibitor dostarlimab as a monotherapy or in combination with radiotherapy and chemotherapy in patients with dMMR-type LACRC. The study included 16 patients with dMMR LACRC, all of whom received six months of dostarlimab monotherapy. Of the 12 patients who completed the treatment, all achieved a complete clinical response (cCR) and did not require radiotherapy, chemotherapy, or surgery. During the 6 to 25 months of follow-up, no disease progression or recurrence was observed, and there were no reports of adverse events of grade 3 or above [88]. The study suggests that following neoadjuvant immunotherapy, patients may consider a watch-and-wait strategy, avoiding potential negative impacts on fertility, sexual health, as well as bowel and bladder function that may result from radiotherapy, chemotherapy, or surgery. Given the increasing incidence of rectal cancer in young patients, this study provides a new treatment option for individuals of childbearing age [89].

The SYSUCC study is a phase II clinical trial evaluating the use of sintilimab in treating dMMR/MSI-H CRC. The study confirms that PD-1 monoclonal antibody immunotherapy can lead to cCR in patients with locally advanced dMMR/MSI-H CRC, thus avoiding the need for radiotherapy, chemotherapy, and surgery while preserving organ function, fundamentally altering the treatment strategy for this disease [82].

In a phase Ib, randomized, and open-label study, the combination therapy of IBI310 and sintilimab was applied as neoadjuvant treatment for operable CRC patients with dMMR/MSI-H characteristics, and significant therapeutic effects were achieved. The core objective of this study was to explore the potential of dual immunotherapy in improving OS in patients. The research data revealed that when IBI310 was used in combination with sintilimab, the pathological complete response rate reached 80%, which was twice the rate of the group treated with sintilimab alone [90]. These findings not only highlight the significant advantage of dual immunotherapy in efficacy but also provide a new treatment option for patients, which may spare them from surgery and thus have broad prospects for clinical application.

A study evaluated the efficacy of tislelizumab as neoadjuvant therapy in patients with locally advanced dMMR/MSI-H CRC [91]. The study enrolled 33 patients aged ≥ 18 years with stage II/III disease, Eastern Cooperative Oncology Group Performance Status 0–1, and eligible for complete resection. Patients received tislelizumab 200 mg intravenously every three weeks for 3 cycles, followed by surgical resection within 10 weeks after the first dose of tislelizumab. Among the 29 patients who underwent surgery, the MPR rate was 89.7% and the pCR rate was 62.1%. The study results support the importance of MSI and MMR testing in the treatment of CRC, and demonstrate that tislelizumab achieves high MPR and pCR rates in patients with dMMR/MSI-H CRC, indicating significant anti-tumor activity and favorable surgical outcomes (Table 2).

**Table 2:** Key clinical trials of neoadjuvant therapy in dMMR/MSI-H CRC

Clinical trial	Interventions	Stage	Treatment duration	Enrollment	pCR (%)	MPR (%)
NICHE-1	nivolumab + ipilimumab	Stage I-III CRC	6 weeks	20	60%	95%
NICHE-2	nivolumab + ipilimumab	Locally advanced CRC	6 weeks	111	68%	95%
NICHE-3	nivolumab +relatlimab	Locally advanced dMMR CRC	4 weeks	19	79%	89%
PICC	toripalimab ± COX-2	Locally advanced CRC	3 months	17 vs. 17	88% vs. 65%	88% vs. 65%
MDACC	pembrolizumab	Locally advanced solid tumor	6 months to 1year	12	83%	92%
NCT05890742	sintilimab ± IBI310	Locally advanced resectable MSI-H CRC	6weeks	52 vs. 49	80% vs. 47.7%	-
NCT05116085	tislelizumab	Stage II-III dMMR/MSI-H CRC	10weeks	33	18/29(62.1%)	26/29(89.7%)
MSKCC	dostarlimab	LACRC	6 months	14	100% (cCR)	-
SYSUCC	sintilimab	LACRC	6 months	16	75% (cCR/CR)	-

MSI-H: microsatellite instability high, LACRC: locally advanced colorectal cancer, pCR: pathologic complete response, MPR: major pathological response, cCR: complete clinical response, CR: complete response

### Adjuvant therapy

Patients with dMMR/MSI-H CRC may benefit from adjuvant therapy using oxaliplatin-based regimens following curative surgery. However, a pooled analysis of four phase III trials revealed that dMMR/MSI-H CRC patients had significantly poorer progression-free survival (PFS) () and OS after standard first-line chemotherapy compared to pMMR/MSS CRC patients. This can be attributed to the high mutational burden of dMMR/MSI-H CRC tumors, which makes them responsive to immunotherapy but less responsive to certain chemotherapeutic agents. As a result, according to NCCN and CSCO guidelines, the use of fluoropyrimidine monotherapy for adjuvant therapy in stage II MSI-H CRC patients is not recommended [92].

### First-line treatment

In the era of immunotherapy, the use of immune treatment in the first-line, early neoadjuvant, and adjuvant therapy for dMMR/MSI-H CRC patients has expanded. In 2020, the FDA approved pembrolizumab as a first-line treatment for unresectable or mCRC with dMMR/MSI-H [93]. The KEYNOTE-177 study showed that compared to chemotherapy, the median PFS of the pembrolizumab treatment group was longer (16.5 months vs. 8.2 months) and the ORR increased by 10.7%, confirming its lasting anti-tumor effects and good safety [83], Given its high efficacy and low toxicity, pembrolizumab was approved by the FDA in June 2020 for first-line treatment of dMMR/MSI-H mCRC [94, 95].

In the phase II multi-cohort CheckMate142 study [96], nivolumab monotherapy or in combination with ipilimumab for the treatment of mCRC patients demonstrated good efficacy and safety. The study showed a single-agent ORR of 34% for second-line and above treatments, and a combined treatment ORR of 65%; for first-line monotherapy, the ORR was 69%, with a disease control rate (DCR) of 84%. The median duration of response (DOR), PFS, and OS were not reached, and the 2-year PFS rate and OS rate were 74% and 79% respectively. The results suggest that dMMR/MSI-H LACRC patients can benefit from dual immunotherapy across all lines [97–99].

In the randomized phase III CheckMate 8HW study, the combination of nivolumab and ipilimumab significantly improved PFS in first-line treatment of dMMR/MSI-H mCRC compared to chemotherapy. At a median follow-up of 24.3 months, the PFS for the nivolumab + ipilimumab group exceeded 38 months, with a 2-year PFS rate of 72%, while the median PFS for the chemotherapy group was 5.9 months. The risk of disease progression or death was reduced by 79%. The study results support nivolumab + ipilimumab as a new standard for first-line treatment of dMMR/MSI-H mCRC patients [98].

### Second and subsequent line of treatments

The results of the KEYNOTE-016 study, presented at the 2015 ASCO Annual Meeting, demonstrated the benefit of single-agent pembrolizumab treatment in patients with dMMR mCRC, marking the beginning of the era of immunotherapy for CRC [100].

KEYNOTE-164 further confirmed the efficacy and manageable safety profile of pembrolizumab in dMMR/MSI-H CRC patients [101, 102]. In 2017, both pembrolizumab and nivolumab received FDA approval for use in dMMR/MSI-H mCRC. The recommendations for CRC immunotherapy mainly apply to dMMR/MSI-H patients. Updated results from the CheckMate142 study demonstrated a 12-month PFS of 77% and OS of 84% in first-line dual immunotherapy. Nivolumab provides durable relief and disease control in pretreated dMMR/MSI-H mCRC patients, potentially establishing a novel treatment avenue for this patient population [103].

PRODIGE54-SAMCO is a randomized phase II trial that assessed the efficacy and safety of avelumab as a second-line treatment for dMMR/MSI-H mCRC patients. The study found avelumab to be more effective than standard second-line therapies and confirmed that anti-PD-(L)1 monoclonal antibodies retain their efficacy even after first-line treatment, without prior use of immune checkpoint inhibitors. This implies that dMMR/MSI-H mCRC patients should receive ICI therapy as early as possible [104].

Currently approved PD-1/PD-L1 monoclonal antibodies lack phase III randomized controlled clinical trial data for advanced second-line or first-line CRC patients. However, based on the results of phase II clinical studies across various tumor types, some domestically produced PD-1/PD-L1 monoclonal antibodies have obtained domestic indications for the later-line treatment of dMMR/MSI-H solid tumors, such as domestic monoclonal antibodies including envafolimab, serplulimab, tislelizumab, and pucotenlimab [105, 106], as well as imported pembrolizumab and nivolumab (Table 3).

**Table 3:** Key clinical trials of immunotherapy in dMMR/MSI-H CRC

NCT number	Clinical trial	Phase	Status	Population	Arms and Interventions	Enrollment	ORR% (95% CI)	DCR% (95% CI)	PFS% (95% CI)
NCT01876511	Keynote-016	II	Completed	MSI	Cohort A: Pemb10mg/kg Q2W	41	28 m: 54 (37–69) 40 (12–74)	28 m: 80 (65–91) 90 (55–100)	20w: 88 (75–100) 28w: 70 (57–86)
				MSS	Cohort B: Pemb10mg/kg Q2W	25	28 m: 0 (0–14) 0 (0–19)	28 m: 16 (57 − 36) 11 (1–35)	20w: 12 (4–36) 28w: 16 (6–41)
NCT02460198	Keynote-164	II	Completed	dMMR/MSI-H	Cohort A: ≥2 previous systemic treatments, Pemb 200 mg Q3W	61	32.8 (21.3–46.0)	50.8 (37.7–63.9)	2.3 (2.1–8.1)
					Cohort B: ≥1 previous systemic treatments, Pemb 200 mg Q3W	63	34.9 (23.3–48.0)	57.1 (44.0–69.5)	4.1 (2.1–18.9)
NCT02060188	CheckMate-142	II	Active, not recruiting	dMMR/MSI-H	(Nivo 3 mg/ kg + Ipi1mg/kg) Q3W]×4+(Nivo 3 mg/kg)­Q2W	119	55 (45.2–63.8)	12w:79 (70.6–85.9)	9 m:76 (67.0– 87.2);12 m:71 (61.4–78.7)
					(Nivo 3 mg/kg) Q2W	74	31.1 (20.8–42.9)	12w:68.9 (57.1%–79.2)	12 m:50.4 (38.1%– 61.4)
					(Nivo 3 mg/kg) Q2W+(Ipi1mg/ kg)­Q6W	45	69 (53–82)	≥ 12w:84 (70.5–93.5)	12 m:76.4 (60.5– 86.6),18 m:76.4 (60.5–86.6), 24 m: 73.6 (57.2–84.5)
NCT02563002	Keynote-177	III	Completed	153MSI	(Pemb 200 mg) Q3W	153	43.8 (35.8–52.0)	/	12 m:55.3 (47.0–62.9) 24 m: 48.3 (39.9–56.2)
				153MSI	Chemotherapy Q2W	154	33.1 (25.8–41.1)	/	12 m:37.3 (29.0–45.5) 24 m:18.6 (12.1–26.3)

MSI: microsatellite instability, MSS: microsatellite stability, dMMR: mismatch repair deficiency, MSI-H: microsatellite instability high, Pemb: pembrolizumab, Nivo: nivolumab, Ipi: ipilimumab, ORR: objective response rate, DCR: disease control rate, PFS: progression-free survival

### Other treatments

The Werner Syndrome ATP-dependent Helicase (WRN) enzyme is considered a potential synthetic lethality target for dMMR/MSI-H cancers, showing broad sensitivity, particularly in CRC [107]. A series of studies have elucidated the molecular mechanisms by which MSI-H cancer cells rely on WRN to maintain genomic stability, indicating that the synthetic lethality relationship with WRN arises from the consequences of MMR dysfunction rather than a defect in MMR itself. Research has shown that tumors with WRN mutations exhibit higher TMB, increased PD-L1 expression, and dMMR/MSI-H characteristics compared to wild-type tumors [108]. Although no drugs have yet been approved globally, several WRN inhibitors are in development. VVD-133,214 and HRO761 are two WRN inhibitors showing significant progress [109, 110]. These advancements suggest that WRN inhibitors could offer new therapeutic options for MSI-H cancer patients and may become a strategy in precision medicine [107].

Fecal microbiota transplantation (FMT) involves extracting beneficial microorganisms from the feces of healthy donors and transplanting them into a patient’s gut via endoscopy or capsules to restore microbial balance. A case report in 2018 preliminarily confirmed the potential efficacy of FMT in treating immune-related adverse events [111]. Recent studies have shown that combining FMT with anti-PD-1 therapy significantly improves the median PFS and OS in patients with MSS mCRC [112]. Research also indicates that ginsenoside Rh4 can inhibit CRC progression by modulating gut microbiota balance and bile acid metabolism [113]. Additionally, the presence of Fusobacterium nucleatum (Fn) is closely associated with various pathological features of CRC [114]. Detection of Fn DNA in colorectal tissues has revealed that Fn-positive patients have lower cancer-specific survival and OS, particularly those with Fn strain C2 [115], which dominates the TME and is closely linked to poorer prognosis. In summary, FMT not only plays a role in regulating gut microbiota balance but may also synergize with immunotherapy to enhance CRC treatment outcomes. Furthermore, gut microbiota members such as Fn may serve as biomarkers for prognosis and treatment response in CRC, providing new perspectives and strategies for precision treatment of the disease.

## Prognostic factors

The latest study utilized Cox proportional hazards regression models to analyze the impact of KRAS exon 2 and BRAFV600E mutations on time to relapse (TTR), OS, and survival after relapse (SAR) in MSI-H CRC patients [116]. The results indicated that in dMMR/MSI-H patients, these mutations showed minimal differences in 5-year TTR and OS. However, regardless of MSI status, patients with these mutations had shorter SAR. Additionally, prognostic impact analysis of dMMR/MSI-H mCRC patients receiving immunotherapy revealed that in sporadic CRC and LS patients, LS patients showed a significantly improved PFS, while there was no statistically significant difference in OS improvement, suggesting that LS CRC patients may have a better OS rate [117].

## Directions of future research

Currently, multiple studies are focused on enhancing the efficacy of anti-PD-1 therapy for dMMR/MSI-H mCRC through immune combination therapy. For instance, a ready-made cancer vaccine Nous-209, based on shared neoantigens, has been proven safe and has demonstrated preliminary anti-tumor effects when used in combination with pembrolizumab for dMMR/MSI-H CRC and gastric cancer patients (NCT04041310).

The introduction of immune checkpoint inhibitors has made immunotherapy more attractive and promising for dMMR patients compared to pMMR patients. However, for operable MSI-H CRC, whether neoadjuvant immunotherapy can replace surgery, and whether adjuvant immunotherapy is more beneficial for MSI-H patients than adjuvant chemotherapy, currently lacks definitive conclusions. The potential value of immunotherapy before and after surgery requires further confirmation through phase III clinical studies. Additionally, primary and acquired resistance has been observed in over 50% of dMMR/MSI-H CRC patients and identifying these patients and overcoming resistance will be an important challenge in the future [118]. Currently, there are currently no studies differentiating the treatment differences among LS, LLS, and sporadic CRC types, and the optimal diagnostic criteria for LLS patients have yet to be established. research and targeted therapeutic drug development for various subtypes, as well as the precise stratification of CRC and the innovation of the treatment layout for advanced colon cancer patients, are promising endeavors.

## Data Availability

No datasets were generated or analysed during the current study.

## Abbreviations

CRC: Colorectal cancer
pMMR: Proficient mismatch repair
MSS: Microsatellite stability
dMMR: Mismatch repair deficiency
MSI-H: Microsatellite instability high
LS: Lynch syndrome
LLS: Lynch-like syndrome
MMR: Mismatch repair
MSI: Microsatellite instability
MLH1: Mutl homolog 1
hMLH1: Human mutl homolog 1
MSH2: Muts homolog 2
hMSH2: Human muts homolog 2
MSH6: Muts homolog 6
hMSH6: Human muts homolog 6
PMS2: Postmeiotic segregation increased 2
hPMS2: Human postmeiotic segregation increased 2
EPCAM: Epithelial cell adhesion molecule
MSI-L: Low-level microsatellite instability
HNPCC: Hereditary non-polyposis colorectal cancer
IHC: Immunohistochemistry
BRAF: B-Raf proto-oncogene, serine/threonine kinase
MAPK: Mitogen-activated protein kinase
CIMP-H: High CpG island methylator phenotype
SSA: Sessile serrated adenoma/polyps
PCR: Polymerase chain reaction
NCCN: National Comprehensive Cancer Network
CSCO: Chinese Society of Clinical Oncology
NGS: Next-generation sequencing
b-MSI: Blood-based MSI
ctDNA: Circulating tumor DNA
b-TMB: Blood-based tumor mutational burden
CLR: Crohn’s-like lymphoid reaction
KRAS: Kirsten rat sarcoma
RAS: Rat sarcoma viral oncogene homolog
DFS: Disease-free survival
OS: Overall survival
CR: Complete response
ICI: Immune checkpoint inhibitors
pCR: Pathologic complete response
MPR: Major pathological response
PD-1: Programmed cell death protein 1
PD-L1: Programmed cell death-ligand 1
ORRs: Objective response rates
mCRC: Metastatic colorectal cancer
CTLA-4: Cytotoxic T-lymphocyte-associated protein 4
ESMO: European Society for Medical Oncology
LACRC: Locally advanced colorectal cancer
COX-2: Cyclooxygenase-2
cCR: Complete clinical response
LAG-3: Lymphocyte activation gene-3
DOR: Duration of response
DCR: Disease control rate
PFS: Progression-free survival
SAEs: Serious adverse events
WRN: Werner syndrome ATP-dependent helicase
TME: Tumor microenvironment
TTR: Time to relapse
